# Effects of *Crocus sativus* and its constituent, safranal, and pioglitazone, on systemic inflammation and oxidative stress induced by paraquat aerosol in rats

**DOI:** 10.22038/IJBMS.2024.72996.15867

**Published:** 2024

**Authors:** Arghavan Memarzia, Seydeh Zahra Ghasemi, Fatemeh Amin, Zahra Gholamnezhad, Mohammad Hossein Boskabady

**Affiliations:** 1 Applied Biomedical Research Center, Mashhad University of Medical Sciences, Mashhad, Iran; 2 Department of Physiology, School of Medicine, Mashhad University of Medical Sciences, Mashhad, Iran; 3 Saffron Institute University of Torbat Heydarieh, Torbat Heydarieh, Iran; 4 Physiology‐Pharmacology Research Center, Research Institute of Basic Medical Sciences, Rafsanjan University of Medical Sciences, Rafsanjan, Iran; 5 Department of Physiology and Pharmacology, School of Medicine, Rafsanjan University of Medical Sciences, Rafsanjan, Iran

**Keywords:** Crocus sativus, Inflammation, Oxidative stress, Paraquat, Pioglitazone, Safranal

## Abstract

**Objective(s):**

The effects of *Crocus sativus*, safranal, and pioglitazone on aerosolized paraquat (PQ)-induced systemic changes were examined.

**Materials and Methods:**

Control (Ctrl) and PQ groups of rats were exposed to saline or PQ (27 and 54 mg/m3, PQ-L and PQ-H) aerosols eight times on alternate days. Nine PQ-H groups were treated with dexamethasone (0.03 mg/kg/day, Dexa), two doses of *C. sativus* extract (20 and 80 mg/kg/day, CS-L and CS-H), safranal (0.8 and 3.2 mg/kg/day, Saf-L and Saf-H), pioglitazone (5 and 10 mg/kg/day, Pio-L and Pio-H), and the combination of low dose of the pioglitazone and extract or safranal (Pio + CS and Pio + Saf) after the end of PQ exposure.

**Results:**

Interferon-gamma (INF-γ), interleukin 10 (IL-10), superoxide dismutase (SOD), catalase (CAT), and thiol serum levels were reduced, but tumor necrosis factor (TNF-α), malondialdehyde (MDA), and total and differential WBC were increased in both PQ groups (*P*<0.05 to *P*<0.001). All measured variables were improved in all treated groups (*P*<0.05 to *P*<0.001). The effects of high dose of C. sativus and safranal on measured parameters were higher than dexamethasone (*P*<0.05 to *P*<0.001). The effects of Pio + CS and Pio + Saf treatment on most variables were significantly higher than three agents alone (*P*<0.05 to *P*<0.001).

**Conclusion:**

*C. sativus* and safranal improved inhaled PQ-induced systemic inflammation and oxidative stress similar to those of dexamethasone and showed synergic effects with pioglitazone suggesting the possible PPARγ receptor-mediated effects of the plant and its constituent.

## Introduction

Saffron or *Crocus sativus L* is a traditional plant belonging to the *Iridaceae* family, which falls under the *Liliaceas*. This plant is cultivated in regions with dry and warm climates including Iran, Spain, Greece, Turkey, Azerbaijan, India, Pakistan, and Australia. The carotenoid pigment of saffron flowers influences the yellow or red color of the plant and the name saffron is derived from the Arabic “zafaran” (yellow). *C. sativus *is commonly used as a flavoring and coloring food additive (1, 2). Several therapeutic effects of *C. sativus *and its constituent, safranal were reported in previous studies including the effects on neurodegenerative disorders such as antinociception and effects on cerebral ischemia, Alzheimer’s, and depression (3-5). Also, the effects of the plant and its bio-active compounds on cancer (6), microbial infections (7), urinary system (8), respiratory (9) coronary artery (10), and gastrointestinal (1) disorders were demonstrated. The effects of *C. sativus* could be related to its ingredients such as crocins, crocetin, picrocrocin, and safranal (1). Therefore, phenolic compounds such as flavonoids and anthocyanins may be biologically active ingredients of the plant (4). The effect of *C. sativus *and its constituents on inflammation and immune dysregulation through modulation of inflammatory cytokines, oxidative stress factors, and immune markers was shown (11-13). Regarding the effect of *C. sativus *and safranal on the respiratory system, the relaxant effect of the extract of the plant on tracheal smooth muscle was reported (9). The effect of the extract of *C. sativus *and safranal, on lung pathology and lung inflammation (11) and the effect of extract of the plant on tracheal responsiveness and plasma levels of IL-4, IFN-γ, total NO, and nitrite (13) in a guinea-pig model of asthma were shown. 

Peroxisome proliferation activating receptors (PPARs) are nuclear receptors that mediate various types of metabolism pathways such as β-oxidation of fatty acids and lipid synthesis. PPAR-γ recapitulates the cellular and molecular pathways determining normal cell structure and function and has potent anti-inflammatory effects (14). Activation of the PPAR-γ receptor blocks the expression of transforming growth factor (TGF) and inhibits the levels of inflammatory cytokines (15). PPAR-γ modulates allergic inflammation through up-regulation of phosphatase and tensin homolog (PTEN) (16). The combination effects of other natural products and their components with PPAR-gamma agonist, pioglitazone, on PQ-induced systemic inflammation and oxidative stress were demonstrated (17).

Agrochemicals such as herbicides and fertilizers showed negative effects on various human organs (17). Pesticide paraquat (PQ) (C12 H14 N2) or bipyridinium poison, is a green liquid that is used in agriculture and is a common agriculture poison with respiratory and systemic toxic effects on humans and animals. This toxic agent could be absorbed via the digestive system, skin, and pulmonary system (18), and 2000 deaths were reported in Japan due to PQ digestion (1). PQ exposure causes multi-organ damage such as swelling, bleeding, inflammation, epithelial cell proliferation, abdominal pain, loss of appetite, nausea, vomiting, diarrhea, systemic inflammation, and mortality due to this agent depending on its concentration in the blood (19). The risk of inflammation, oxidative stress, and immune dysregulation due to PQ exposure via increasing inflammation markers, oxidative factors, and immune response were indicated (17, 18). In this study, the effect of *C. sativus*, its constituent, saffron, and pioglitazone alone and their combination on systemic inflammation and oxidative stress induced by inhaled PQ were examined in rats.

## Materials and Methods

In this study, 84 male Wistar rats weighing 200±20 g were obtained from the Animal House, School of Medicine, Mashhad University of Medical Sciences, Mashhad (MUMS), Iran, and kept at 22±2 °C, with 12 hr light/dark cycles. Animals had free access to standard diet and tap water. The study was approved by the Ethics Committee of MUMS for animal studies (Code 970738). All experiments on animals were performed in accordance with national laws regarding the care and use of laboratory animals. 

***Animal grouping***

The study was done on 12 groups of rats (n = 7 in each group) including the control (Ctrl) group which were exposed to saline aerosols. Two groups were exposed to PQ aerosol (27 and 54 mg/m3, PQ-L and PQ-H). Nine PQ-H groups were treated with dexamethasone (0.03 mg/kg/day, Dexa), two doses of *C. sativus *extract (20 and 80 mg/kg/day, CS-L and CS-H, respectively) and safranal (0.8 and 3.2 mg/kg/day, Saf-L and Saf-H, respectively), pioglitazone (5 and 10 mg/kg/day, Pio-L and Pio-H, respectively), and the combination of low dose of the pioglitazone with extract or safranal (Pio + CS and Pio + Saf) after the end of PQ exposure. The details of the groups are shown in Table 1.

***Preparation of blood sample***

At the end of the treatment period (day 32), the rats were anesthetized using ketamine (80 mg/kg) and xylazine (10 mg/kg), and blood samples (5 ml per/rat) were taken out by cardiac puncture. One milliliter was kept in an anticoagulant-containing tube for examining total white blood cells (WBC) and the remaining was centrifuged at 2000 revolutions per minute (rpm) for 10 min. 

***Total and differential WBC determination***

 Using a Neubauer chamber, total WBC was counted in duplicate, and differential WBC was counted by preparing a blood smear stained with Wright–Giemsa as described previously (20).

***Oxidant and anti-oxidant markers determination***

The levels of oxidant and anti-oxidant markers including malondialdehyde (MDA), and thiol as well as catalase (CAT) and superoxide dismutase (SOD) activities were measured in the serum as previously described (21). Briefly, for MDA measurement, 2 ml reagent of TBA/trichloroacetic acid (TCA)/HCl was added to 1 ml of serum and heated in a water bath for 40 min, cooled, and centrifuged at 1,000×g for 10 min, The absorbance was measured at 535 nm and MDA concentration (C) in nM was calculated as C = Absorbance/ (1.56×105). 

The activity of SOD was measured by generation of superoxide by pyrogallol auto-oxidation and the inhibition of superoxide-dependent reduction of the tetrazolium dye, MTT (3-(4, 5-dimethylthiazol-2-yl, 2, 5-diphenyltetrazolium bromide) to its formazan by SOD and was measured at 570 nm and expressed as unit (U)/ml. 

The activity of CAT was estimated by determination of the rate constant, k, (dimension: s-1, k) of hydrogen peroxide decomposition using determination of the reduction in absorbance at 240 nm/min and expressed as unit (U)/ml (22). 

Total thiol concentration was measured by adding 1 ml trisethylene diamine tetraacetic acid (EDTA) buffer to 50 μl serum in 1 ml cuvettes and read at 412 nm against Tris-EDTA buffer alone (A1). Then, 20 μl DTNB reagents were added to the mixture and kept for 15 min and the sample absorbance was read again (A2). The absorbance of the DTNB reagent was read as a blank (B) and the following equation was used to calculate total thiol concentration (mmol/l):

Total thiol concentration (mmol/l) = (A2–A1–B)×1.07/0.05×13.6.

***Cytokines measurements***

Cytokine levels including IL-10, IFN-γ, and TNF-α were measured in the supernatant of serum using specific enzyme-linked immunosorbent assay (ELISA) kits (Karmania Pars, Kerman, Iran) according to the manufacturer’s technique and previous reports (23). 

***Statistical analysis***

The results were presented as mean±SEM. One-way analysis of variance (ANOVA) followed by Tukey’s multiple comparison test was employed for the comparison of the data. Significance was accepted at the level of *P*<0.05.

## Results

***Oxidant and anti-oxidants levels***

The serum level of MDA was increased but the activities of antioxidant enzymes including SOD and CAT as well as thiol level were decreased in exposed groups to both doses of PQ compared to the control group except for SOD in the PQ-L group (*P*<0.01 to *P*<0.001). Treatment with both doses of C. sativus extracts, safranal, pioglitazone and their combination (Pio + CS and Pio + Saf), and dexamethasone increased SOD and CAT activities and thiol level but reduced MDA level in the serum compared to the PQ-H except CAT activity in CS-L, CS-H and Pio-L groups and SOD activity and thiol level in Pio-L group (*P*<0.05 to *P*<0.001). The effects of high dose of the extract, safranal, and pioglitazone on MDA and SOD, and the effects of high dose of safranal and pioglitazone on thiol level were significantly higher than their low dose (*P*<0.05 to *P*<0.01) (Figures 2 and 3). The effects of treatment with dexamethasone on MDA level were higher than Pio-L, Pio-H, and CS-L on thiol level was higher than Pio-L, and CS-L on CAT activity was higher than Pio-L, but its effect was lower on both MDA and thiol than Saf-H and on SOD was lower than Saf-H and Pio + Saf (*P*<0.05 to *P*<0.001) (Figures 2 and 3). The effect of the combination treatment of Pio + CS and Pio + Saf on oxidative stress markers was significantly higher than the three agents alone (*P*<0.05 to *P*<0.001), except differences between Pio + CS vs CS-L for CAT and Pio + CS vs Pio-L for SOD (Figures 2 and 3).

***Total and diff white blood cells (WBCs) counts in serum***

Total and differential WBC counts in the blood were increased following animal exposure to both doses of PQ compared to the control group except for monocyte in the PQ-L group (*P*<0.01 to *P*<0.001). Treatment with both doses of *C. sativus *extract, safranal, pioglitazone, the combination of Pio + CS and Pio + Saf, and dexamethasone decreased total and differential WBCs counts except monocyte count in the CS-L, Saf-L, Pio-L and Pio + CS groups and eosinophil in the CS-L group (*P*<0.05 to *P*<0.001) (Figures 4 and 5). The effects of high dose of the extract, safranal, and pioglitazone on total WBC, eosinophils, and lymphocytes, the effects of high dose of safranal and pioglitazone on eosinophils and lymphocytes, and the effect of high doses of pioglitazone on monocytes were significantly higher than their low dose (*P*<0.05 to 0.001) (Figures 4 and 5). The effects of CS-L and Pio-L on total WBC, the effects of all treatment groups except Saf-H and Pio + Saf on neutrophils, the effects of Pio-H and Pio + Saf on eosinophils and Saf-H, Pio-H, and Pio + Saf on lymphocytes, and the effects of all treated groups on monocytes were significantly lower than those of dexamethasone. However, the effect of Saf-H on total WBC was higher than the effect of dexamethasone (*P*<0.05 to *P*<0.001) (Figures 4 and 5). The effects of Pio + CS and Pio + Saf on total and differential WBC counts were significantly higher than those of the three agents alone. In addition, the effects of Pio + Saf on neutrophil and lymphocyte counts were significantly higher than those of Pio-L and Saf-L groups but the effects of Pio + CS on neutrophil were only higher than the effects of Pio-L, and on lymphocyte counts were higher than the effect of CS-L. The effect of only Pio + Saf on monocyte count was higher than the Pio-L group (*P*<0.05 to *P*<0.001) (Figures 4 and 5).

***Cytokines levels in serum***

Serum levels of INF-γ and IL-10 were decreased but TNF-α level was increased following administration of two doses of PQ (27, 54 mg/m3) compared to the control group (*P*<0.05 to *P*<0.01). The levels of INF-γ and IL-10 were significantly increased but TNF-α level was reduced in all treated groups except IL-10 level in treated groups with low dose of safranal and pioglitazone (*P*<0.05 to *P*<0.001). The effects of the high dose of the extract, safranal, and pioglitazone on all measured cytokines were significantly lower than their low dose (*P*<0.05 to *P*<0.001). The effect of dexamethasone on INF-γ level was higher than CS-L, Saf-L, and Pio-L and on TNF-α than Saf-L and Pio-L. However, the effect of dexamethasone on TNF-α was lower than in CS-H, Saf-H, and Pio + CS groups (*P*<0.05 to *P*<0.001). The treatment effects of Pio + CS and Pio + Saf combination groups were higher than tree agents alone except for the differences between Pio + CS vs CS-L and Pio + Saf vs Saf-L for IL-10 (*P*<0.05 to *P*<0.001) (Figure 6).

**Table 1:** Various animal groups of the present study

No.	Abbreviations	Group	Aerosol exposure	Treatment
1	Ctrl	Control	Salin aerosol	-
2	PQ-L	Paraquat	PQ, 27 mg/m^3^	-
3	PQ-H		PQ, 54 mg/m^3^	-
4	CS-L	*C. sativus*	“	20 mg/kg/day
5	CS-H		“	80 mg/kg/day
6	Saf-L	Safranal	“	0.8 mg/kg/day
7	Saf-H		“	3.2 mg/kg/day
8	Pio-L	Pioglitazone	“	5 mg/kg/day
9	Pio-H		“	10 mg/kg/day
10	Pio + CS	Combination treated	“	5 + 20 mg/kg/day
11	Pio + Saf		“	5 + 0.8 mg/kg/day
12	Dexa	Dexamethasone	“	0.03 mg/kg/day

Animals were exposed to saline in control (Ctrl) group and to paraquat (PQ) aerosols in the other groups 8 times during 15 days (i.e. on days 1, 3, 5, 7, 9, 11, 13 and 15), each time for 30 min by inhalation as previously described (22). PQ groups were treated with saline, 20 and 80 mg/kg/day hydro-alcoholic extract of* C. sativus* (CS-L and CS-H), 0.8 and 3.2 mg/kg/day safranal (Saf-L and Saf-H), (11, 13) 5 and 10 mg/kg/day pioglitazone (Pio-L and Pio-H), combination of low dose of the pioglitazone with the extract or safranal (Pio + CS and Pio + Saf) and dexamethasone (Dexa) for 16 days after the end of PQ exposure period (20), (n = 7 in each group). Pioglitazone was injected intraperitoneally (IP), but the extract, safranal and dexamethasone were administered by gavage similar to a previous study (24).

**Figure 1:**
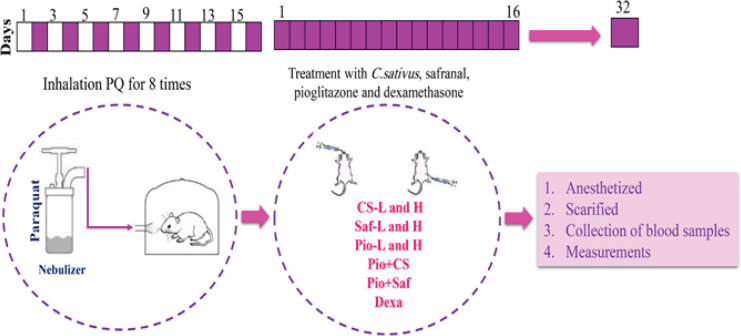
Time table of PQ exposure and treatment in different studied groups

PQ paraquat, CS-L and CS-H, Saf-L and Saf-H, Pio-L and Pio-H, Pio + CS, Pio + Saf and Dexa are groups exposed to PQ-H and treated with 20 and 80 mg/kg/day extract, 0.8 and 3.2 mg/kg/day safranal, 5 and 10 mg/kg/day pioglitazone, Pio, 5 + CS, 20 mg/kg/day, Pio, 5 + Saf, 3.2 mg/kg/day and 0.03 mg/kg/day dexamethasone, respectively.

PQ: Paraquat

**Figure 2:**
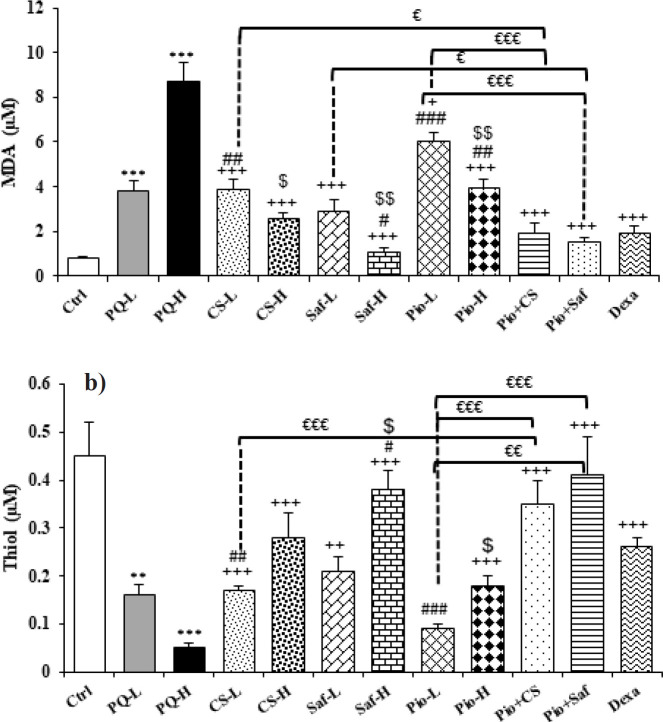
Serum levels of MDA (a) and thiol (b) in different studied groups

The results are expressed as mean ± SEM (n = 7 in each group). ** *P*<0.01 and *** *P*<0.001 compared to the control group. + *P*<0.05, ++ *P*<0.01 and +++ *P*<0.001 compared to the PQ-H group. # *P*<0.05, ## p<0.01 and ### *P*<0.001 compared to dexamethasone treated group. € *P*<0.05, €€ *P*<0.01 and €€€ p<0.001 compared to the combination treated groups. $ *P*<0.05 and $$ *P*<0.01 compared to low doses of the extract, safranal and pioglitazone. Comparisons between different groups were made using one-way ANOVA followed by Tukey’s multiple comparison test. Ctrl, control group, PQ-L and PQ-H, groups exposed to 27 and 54 mg/m^3^ paraquat aerosols, CS-L, CS-H, Saf-L, Saf-H, Pio-L, Pio-H, Pio + CS, Pio + Saf and Dexa are groups exposed to PQ-H and treated with 20 and 80 mg/kg/day extract, 0.8 and 3.2 mg/kg/day safranal, 5 and 10 mg/kg/day pioglitazone, Pio,5 + CS, 20 mg/kg/day, Pio, 5 + Saf, 0.8 mg/kg/day and 0.03 mg/kg/day dexamethasone, respectively.

MDA: malondialdehyde; PQ: paraquat

**Figure 3:**
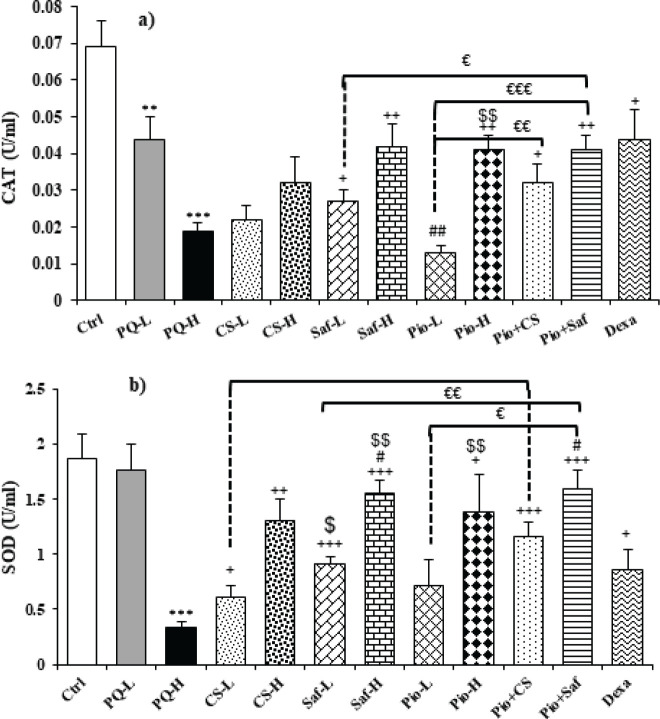
Serum activities of SOD (a) and CAT (b) in different studied groups

The results are expressed as mean ± SEM (n = 7 in each group). ** *P*<0.01 and *** *P*<0.001 compared to the control group. + *P*<0.05, ++ *P*<0.01 and +++ *P*<0.001 compared to the PQ-H group. # *P*<0.05 and ## *P*<0.01 compared to dexamethasone treated group. € *P*<0.05, €€ *P*<0.01 and €€€ *P*<0.001 compared to the combination treated groups. $ *P*<0.05 and $$ p<0.01 compared to low doses of the extract, safranal and pioglitazone. Comparisons between different groups were made using one-way ANOVA followed by Tukey’s multiple comparison test. Ctrl, control group, PQ-L and PQ-H, groups exposed to 27 and 54 mg/m^3^ paraquat aerosols, CS-L, DS-H, Saf-L, Saf-H, Pio-L, Pio-H, Pio + CS-, Pio + Saf and Dexa are groups exposed to PQ-H and treated with 20 and 80 mg/kg/day extract, 0.8 and 3.2 mg/kg/day safranal, 5 and 10 mg/kg/day pioglitazone, Pio,5 + CS, 20 mg/kg/day, Pio, 5 + Saf, 0.8 mg/kg/day and 0.03 mg/kg/day dexamethasone respectively.

**Figure 4:**
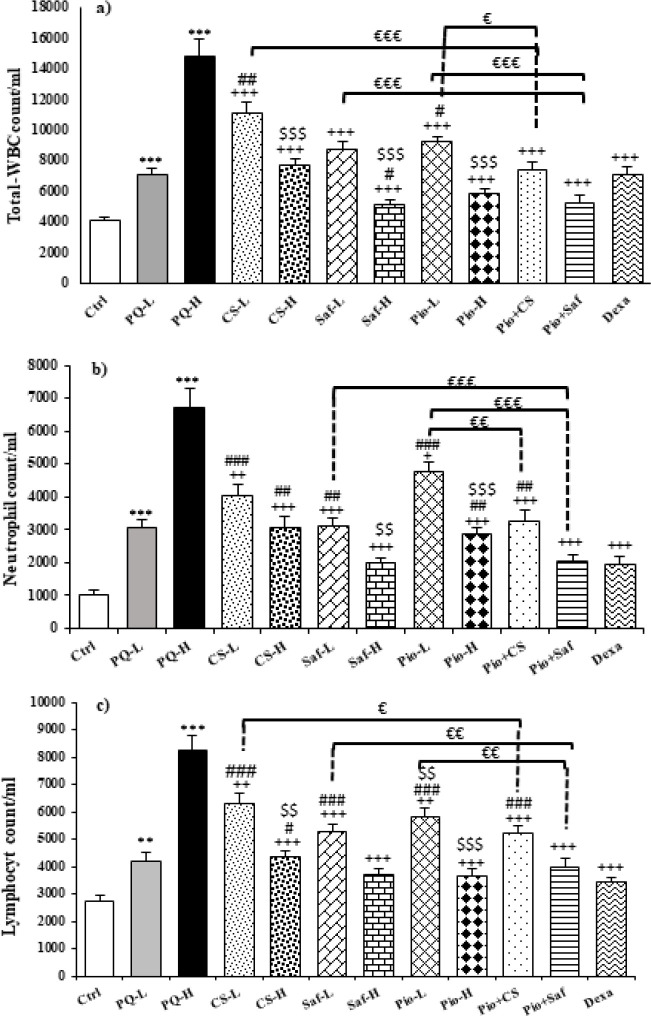
Total WBCs (a), neutrophil (b) and lymphocyte (c) counts in the blood of different studied groups

The results are expressed as mean ± SEM (n = 7 in each group). *** p<0.001compared to the control group. + *P*<0.05, ++ *P*<0.01 and +++ *P*<0.001 compared to the PQ group. # *P*<0.05, ## *P*<0.01 and ### *P*<0.001 compared to dexamethasone treated group. € *P*<0.05, €€ *P*<0.01 and €€€ *P*<0.001 compared to combination treated groups. $ *P*<0.05, $$ *P*<0.01, and $$$ p<0.001 compared to low doses of the extract, safranal and pioglitazone. Comparisons between different groups were made using one-way ANOVA followed by Tukey’s multiple comparison test. Ctrl, control group, PQ-L and PQ-H, groups exposed to paraquat aerosol with dose of 27, 54 mg/m^3^, CS-20,80 mg/kg/day, , CS-L, DS-H, Saf-L, Saf-H, Pio-L, Pio-H, Pio + CS-, Pio + Saf and Dexa are groups exposed to PQ-H and treated with 20 and 80 mg/kg/day extract, 0.8 and 3.2 mg/kg/day safranal, 5 and 10 mg/kg/day pioglitazone, Pio,5 + CS, 20 mg/kg/day, Pio, 5 + Saf, 0.8 mg/kg/day and 0.03 mg/kg/day dexamethasone, respectively.

**Figure 5:**
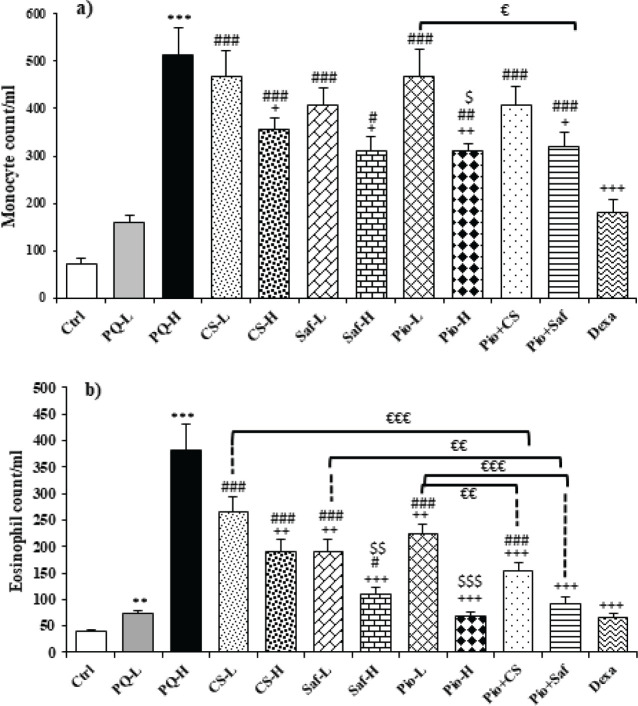
Monocyte (a) and eosinophil (b) counts in blood in different studied groups

The results are expressed as mean ± SEM (n = 7 in each group). ** *P*<0.01 and *** *P*<0.001compared to the control group. + *P*<0.05, ++ *P*<0.01 and +++ *P*<0.001 compared to the PQ group. ### p<0.001 compared to dexamethasone treated group. € *P*<0.05, €€ *P*<0.01 and €€€ *P*<0.001 compared to combination treated groups. $ *P*<0.05, $$ *P*<0.01 and $$ *P*<0.01 compared to low doses of the extract, safranal and pioglitazone. Comparisons between different groups were made using one-way ANOVA followed by Tukey’s multiple comparison test. Ctrl, control group, PQ-L and PQ-H, groups exposed to paraquat aerosol with dose of 27, 54 mg/m^3^, CS-20,80 mg/kg/day, , CS-L, DS-H, Saf-L, Saf-H, Pio-L, Pio-H, Pio + CS-, Pio + Saf and Dexa are groups exposed to PQ-H and treated with 20 and 80 mg/kg/day extract, 0.8 and 3.2 mg/kg/day safranal, 5 and 10 mg/kg/day pioglitazone, Pio,5 + CS, 20 mg/kg/day, Pio, 5 + Saf, 0.8 mg/kg/day and 0.03 mg/kg/day dexamethasone, respectively.

**Figure 6:**
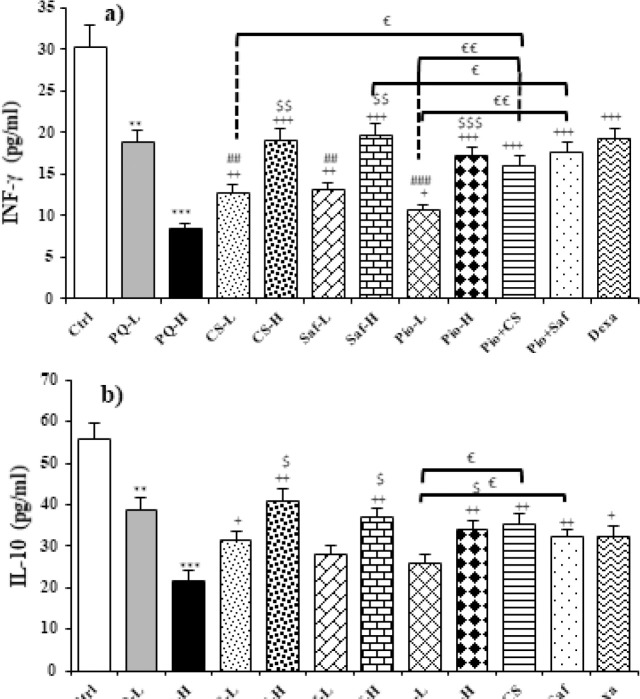
Serum levels of INF-γ (a), IL-10 (b), and TNF-α (c), in different studied groups

The results are expressed as mean ± SEM (n = 7 in each group). * *P*<0.05, ** *P*<0.01 and *** *P*<0.001compared to the control group. + *P*<0.05, ++ *P*<0.01 and +++ *P*<0.001 compared to the PQ group. # *P*<0.05, ## *P*<0.01 and ### *P*<0.001 compared to dexamethasone treated group. € *P*<0.05, €€ *P*<0.01 and €€€ *P*<0.001 compared to combination treated groups. $*P*<0.05, $$ *P*<0.01 and $$ *P*<0.01 compared to group low doses of pioglitazone and the extract, safranal. Comparisons between different groups were made using one-way ANOVA followed by Tukey’s multiple comparison test. Ctrl, control group, PQ-L and PQ-H, groups exposed to paraquat aerosol with dose of 27, 54 mg/m3, CS-20,80 mg/kg/day, , CS-L, DS-H, Saf-L, Saf-H, Pio-L, Pio-H, Pio + CS-, Pio + Saf and Dexa are groups exposed to PQ-H and treated with 20 and 80 mg/kg/day extract, 0.8 and 3.2 mg/kg/day safranal, 5 and 10 mg/kg/day pioglitazone, Pio,5 + CS, 20 mg/kg/day, Pio, 5 + Saf, 0.8 mg/kg/day and 0.03 mg/kg/day dexamethasone, respectively.

## Discussion

This study was designed to evaluate the treatment effects of C. sativus extract, safranal, pioglitazone, and the combination of pioglitazone + the extract and safranal on systemic inflammation and oxidative stress induced by PQ aerosol in rats. The levels of oxidants and antioxidants, total and differential WBC counts, and cytokine levels including INF-γ, IL-10, and TNF-α were measured in the blood of the studied groups. According to the results of this study, exposing rats to PQ with 27 and 54 mg/m3 doses 8 times on alternative days, increased MDA level as an oxidant marker but decreased thiol levels s SOD and CAT activities as antioxidant parameters, increased total and differential WBC counts, decreased the levels of anti-inflammation cytokines including INF-γ and IL-10 but increased inflammation markers such as TNF-α in the blood. Therefore, the results of this study demonstrated systemic inflammation and oxidative stress induced by PQ aerosols in rats.

The relevant studies reported that administration of PQ aerosols (54 mg/m^3^) for 8 days in rats increased total and differential WBC counts, NO, MDA, IL-10, and INF-γ but decreased thiol level, SOD, CAT activities, IL-17, and TNF-α levels in serum (17). It was indicated that the serum levels of antioxidant activities including SOD and CAT and thiol levels were decreased but the level of MDA and WBC numbers were increased in the blood following PQ inhalation in rats (24). Inhalation of PQ aerosols (54 mg/m^3^) in Wistar rats significantly enhanced MDA and nitrite (NO) levels but reduced IFN-γ/IL-6 ratio as well as CAT activities and IFN-γ level in serum in a rat model (18). Another study reported that serum levels of cytokines including IL-6 were increased but IFN-γ was decreased in rats after PQ (54 mg/m^3^) administration for 16 days (25). The above studies supported the induction of systemic oxidative stress and inflammation by PQ administration in different models and supported the findings of the present study.

Treatment with 20 and 80 mg/kg/day extract, 0.8 and 3.2 mg/kg/day safranal, 5 and 10 mg/kg/day pioglitazone and dexamethasone as well as combination of low dose of pioglitazone with extract or safranal for 16 days after the end of PQ exposure increased SOD and CAT activities, also INF-γ and IL-10 levels but reduced MDA and TNF-α levels as well as total and differential WBC counts in the blood.

Previous studies indicated that treatment with *C. sativus *extract and safranal improved total and differential WBC counts in the blood and the BALF, histamine level, and lung pathological changes, in an asthma model of rats (11). Administration of *C. sativus* (40 μg/ml) reduced oxidative stress and carbonyl stress via reactive oxygen species (ROS) in the diabetes mellitus model (26). Also, it was reported that safranal significantly inhibited the ROS generation and cell apoptosis induced by rotenone as well as toxicity and inflammation via inhibition of kelch-like ECH-associated protein 1 (Keap1) and promoted the nuclear translocation of nuclear factor erythroid 2-related factor 2 (Nrf2) expression in rotenone-induced dopaminergic neurons in a Parkinson’s disease model (27). Treatment of streptozotocin-induced animal model of diabetes with safranal (0.25, 0.50, and 0.75 mg/kg/day, IP for 4 weeks) reduced MDA, NO, and glutathione (GSH) contents but increased the activities of SOD and CAT in the BALF and lung tissue of diabetic rats (28). The anti-inflammatory, antioxidant, and immunological effects of saffron and its constituents were described in a review article (12). All of the above studies support the finding of the current study regarding the treatment effect of *C. sativus* and safranal on systemic inflammation and oxidative stress induced by inhaled PQ.

The anti-inflammatory and antioxidant effects of pioglitazone and other PPAR-γ agonists are well documented (29). Pioglitazone also improved antioxidant capacity, and increased SOD and CAT activities in a kidney ischemia-reperfusion model. Increased IL-4, but decreased IFN-γ, TNF-α, and IL-6 levels were reported by pioglitazone (30). Treatment with a combination of pioglitazone (15 mg/kg/day) and metformin improved lung adenoma (31). Pioglitazone (10 μM, for 1 or 3 hr) treatment also decreased degranulation and adhesion of neutrophils in LPS-induced lung injury (22). The result of other studies showed that pioglitazone decreased oxidants, IL-10, IL-6, total and differential WBC counts, increased antioxidant activities, IL-17, TNF-α, IFN-γ, and IFN-γ/IL-6 ratio (17, 23). These studies support the effects of pioglitazone on systemic inflammation and oxidative stress due to inhaled PQ in rats observed in the current study. The improvement effects of the combination of pioglitazone with other extracts and constituencies such as *Zataria multiflora* extract and carvacrol on PQ inducing model were reported by previous studies (17, 18, 23).

The results of this study also indicated similar results of the extract and safranal with dexamethasone as a positive control. The effects of a low dose of the extract and safranal on some measured values were lower but the effects of their high dose on most values were higher than dexamethasone. The similar effects of *C. sativus *and safranal with dexamethasone on PQ-induced systemic changer, support the anti-inflammatory and antioxidant properties of saffron and its constituent, safranal.

The effects of combinations of Pio + CS-L and PIO + Saf-L were evaluated to determine the synergic (additive) effects of pioglitazone with the extract or safranal. In the combination-treated groups with low doses of Pio + CS or Saf, the improvements of almost all measured variables were significantly higher than the three agents alone. The synergic effects of the combination of pioglitazone and other herbal extracts and their constituents on systemic inflammation and oxidative damage were indicated in previous studies (17, 23, 24). These results showed synergic effects of the plant and its constituent safranal with pioglitazone which suggests PPAR-γ receptor-mediated effects of saffron and safranal. However, this suggestion should be confirmed by the effect of the plant and safranal in the presence of a PPAR-γ receptor antagonist against PQ-induced systemic oxidative stress and inflammation in further studies. 

In the present study, the effects of two doses of PQ, pioglitazone, extract, and safranal were examined. However, it is recommended that in further study(s) the effects of three doses of each agent be evaluated.

## Conclusion

*C. sativus *and safranal ameliorated systemic inflammation and oxidative stress induced by PQ aerosol which was similar to the effects of PPAR-γ agonist, pioglitazone, and dexamethasone. In addition, C. sativus and safranal showed a synergic effect with pioglitazone which suggests PPAR-γ receptor-mediated effects of the plant and safranal.

## Authors’ Contributions

All authors contributed to the study’s conception and design. A M, SZ G, F A, and MH B contributed to material preparation, data collection, and analysis. The first draft of the manuscript was written by A M and all authors commented on previous versions of the manuscript. All authors read and approved the final manuscript.

## Financial Disclosure

This work was financially supported by a grant from the Research Council of Mashhad University of Medical Sciences and Ferdowsi University of Mashad, Mashad, Iran (Code: 961202). The results presented in this article are a part of the PhD thesis of Arghavan Memarzia.

## Conflicts of Interest

The authors declare that they have no conflicts of interest.
